# Responsible AI in biotechnology: balancing discovery, innovation and biosecurity risks

**DOI:** 10.3389/fbioe.2025.1537471

**Published:** 2025-02-05

**Authors:** Nicole E. Wheeler

**Affiliations:** Department of Microbes, Infection and Microbiomes, School of Infection, Inflammation and Immunology, College of Medicine and Health, University of Birmingham, Birmingham, United Kingdom

**Keywords:** artificial intellegence, biotechnology, AI safety, protein design and engineering, synthetic biology

## Abstract

The integration of artificial intelligence (AI) in protein design presents unparalleled opportunities for innovation in bioengineering and biotechnology. However, it also raises significant biosecurity concerns. This review examines the changing landscape of bioweapon risks, the dual-use potential of AI-driven bioengineering tools, and the necessary safeguards to prevent misuse while fostering innovation. It highlights emerging policy frameworks, technical safeguards, and community responses aimed at mitigating risks and enabling responsible development and application of AI in protein design.

## Introduction

The convergence of artificial intelligence (AI) and biotechnology is rapidly transforming the landscape of scientific research, promising groundbreaking advancements in medicine, agriculture, and environmental science (OECD, 2023; AI Policy Perspectives, 2024). However, these same tools also present unique biosecurity challenges. The ability of AI to accelerate drug discovery and design novel proteins can, if misused, lower the barriers to developing biological weapons with unprecedented precision and potency (Carter et al., 2023; Sandbrink, 2023; Drexel and Withers, 2024).

This dual-use potential—where innovations designed for beneficial purposes may also enable harm—demands urgent attention from the biotechnology community. This review explores the history and evolving threat model of bioweapons development, outlines specific concerns raised in the light of AI, highlights key mitigations and safeguards already in place, and outlines actionable pathways for biotechnologists to engage with this critical issue. By addressing these risks, the field can ensure that the transformative power of AI is harnessed responsibly, minimising dangers while maximising its potential for good.

### Overview of AI applications in bioscience

AI has driven remarkable advancements in discovery science and deepened our understanding of biological systems, particularly the proteins that underpin essential biological functions. For instance, tools like DeepVariant (Poplin et al., 2018) use AI to identify genetic variants with unprecedented accuracy, aiding genomics research. EVE (Frazer et al., 2021) and AlphaMissense (Cheng et al., 2023) provide valuable insights into the likely impacts of genetic mutations, illuminating the genetic mechanisms underlying disease. Beyond genetic analysis, AlphaFold (Jumper et al., 2021) and RosettaFold (Krishna et al., 2024) have significantly contributed to structural biology by accurately predicting protein 3D structures and complementing experimental methods. Similarly, ESM3 has excelled in protein sequence analysis, helping to bridge the gap between sequence, structure and function (Hayes et al., 2024), and other deep learning models have made further progress helping to address the major scientific challenge of protein function prediction (Bileschi et al., 2022). These tools collectively address one of the central challenges of modern biology: understanding DNA and protein functions and their roles in biological life, marking a new era of AI-enabled scientific discovery.

The integration of artificial intelligence (AI) into bioengineering is helping to transform biological design into a systematic engineering discipline, sparking rapid progress and innovation. AI-driven tools are revolutionising protein design, unlocking opportunities in therapeutics, diagnostics, and synthetic biology. For instance, tools like RFDiffusion (Watson et al., 2023) and Chroma (Ingraham et al., 2023) allow the creation of proteins with desired structures and properties, while DiffDock (Corso et al., 2022) significantly improves the prediction of protein-ligand binding interactions. Tools like DeepBind (Alipanahi et al., 2015) accurately predict protein binding to DNA and RNA, AlphaProteo (Zambaldi et al., 2024) allows the design of novel binders, and AI tools are being used to engineer proteins with greater stability and functionality (Sumida et al., 2024). These innovations tackle long-standing challenges such as engineering enzymes for industrial applications and combating antimicrobial resistance, while also paving the way for transformative advancements in personalised medicine, biomanufacturing, and environmental sustainability. By harnessing the power of AI, bioengineering can strive to achieve levels of precision and reproducibility akin to traditional engineering disciplines, heralding a new era of scientific discovery and application.

AI and robotic scientists represent a cutting-edge application of AI in biotechnology and other scientific fields (OECD, 2023). These systems automate and accelerate the scientific discovery process, from hypothesis generation to experimentation and data analysis. One notable example is Adam, a robot scientist designed to autonomously identify gene functions in yeast, pioneering the integration of AI with laboratory automation (Sparkes et al., 2010). Building on Adam’s success, Eve was developed to accelerate drug discovery and has identified existing compounds with potential applications for treating neglected tropical diseases (Williams et al., 2015). In metabolic engineering, automated systems have shown significant promise in optimizing biological processes. For instance, a study by HamediRad et al. (2019) demonstrated the use of automated tools to enhance experimental success rates and improve yields in the production of valuable biomolecules. Additionally, digital AI systems like the data-to-paper platform (Ifargan et al., 2024), independently analyze large datasets to identify priority findings, providing researchers with actionable insights and reducing time spent on data interpretation.

AI is revolutionizing how scientists generate, process, and disseminate knowledge, fundamentally transforming the research landscape. Its applications span the entire scientific workflow, from gathering and annotating data to modeling complex systems and devising solutions to some of humanity’s most pressing challenges (AI Policy Perspectives, 2024). One striking example of the transformative impact of AI is the recognition of its contributions to protein science through the Nobel Prize in Chemistry. The developers of AlphaFold and Rosetta were awarded the prize for their groundbreaking work in understanding and designing proteins, highlighting how AI tools are enabling researchers to solve problems previously thought insurmountable (Nature, 2024). This recognition underscores the pivotal role AI is playing across disciplines, driving progress not only in protein science but also in areas like climate modeling, materials science, and personalised medicine. By enhancing the speed, scale, and precision of scientific inquiry, AI is unlocking new frontiers of knowledge and reshaping the future of innovation.

### The dual-use dilemma: risks in AI-driven bioengineering

The transformative power of AI in bioengineering comes with inherent risks, particularly due to the dual-use nature of biotechnology—where tools intended for beneficial purposes can also be exploited for malicious ends (National Research Council US, 2007). The incorporation of AI amplifies these concerns by lowering some technical barriers to advanced bioengineering, potentially enabling misuse by malicious actors (Carter et al., 2023; Drexel and Withers, 2024). Historical precedents, such as the development and deployment of biological weapons during the 20th century, underscore the potentially catastrophic consequences of biotechnological misuse.

Recognising these risks, the international community has initiated frameworks such as the Biological Weapons Convention (BWC) (UNODA, 2024) to establish norms against the misuse of biotechnology. Recent efforts, including global AI safety summits, have sought to extend these principles to the intersection of AI and biosecurity. However, these initiatives face significant challenges, including inadequate funding, weak enforcement mechanisms, and the rapid pace of technological advancements (Cropper et al., 2023).

This review delves into the evolving landscape of biosecurity risks associated with AI-powered protein design. It examines the dual-use potential of these tools, evaluates existing and proposed safeguards, and highlights actionable roles for scientists. By addressing these challenges head-on, we can build a secure and resilient ecosystem that maximises the benefits of AI-driven bioengineering while safeguarding against its misuse.

## The changing biorisk landscape

The landscape of biological risks has transformed dramatically over the past century, driven by scientific advancements, changing geopolitical contexts, and the emergence of disruptive technologies like artificial intelligence (AI). Historically, the development and deployment of biological weapons were constrained by significant technical and logistical barriers (Ben Ouagrham-Gormley, 2014; Revill and Jefferson, 2014). However, there are concerns that the growing accessibility of cutting-edge biotechnological tools, particularly those powered by AI, has begun to erode these barriers (Carter et al., 2023; Sandbrink, 2023; Drexel and Withers, 2024). AI-driven applications in synthetic biology, protein design, and genetic engineering have not only accelerated legitimate scientific progress but also expanded the potential for misuse, enabling actors with limited expertise to pursue sophisticated biological capabilities.

This section examines the evolution of biological risks, beginning with the historical context of bioweapons development and the global responses to these threats. It then explores the contemporary challenges posed by the convergence of AI and biotechnology, focusing on how these technologies are reshaping the threat landscape. Finally, it assesses the implications of AI-driven advancements for biosecurity, highlighting the need for proactive measures to address the dual-use nature of these powerful tools.

### Historical context: bioweapons development and challenges

Biological weapons have posed a persistent threat since their initial development in the 20th century. During World War II, several nations invested substantial resources into bioweapons programs, but technical and logistical hurdles often precluded successful deployment. These challenges included the selection of pathogenic strains, difficulties in scaling up production, and ensuring properties such as heat stability and effective dispersal under operational conditions (Ben Ouagrham-Gormley, 2014).

While large-scale bioweapons programs have become less common, sporadic incidents highlight the enduring risks associated with these technologies. Notable examples include the Amerithrax attacks of 2001, where anthrax spores were mailed to targets in the United States, causing public panic and five deaths (Rasko et al., 2011). Another instance occurred in 1984, when a religious commune deliberately contaminated salad bars with *Salmonella* in an Oregon town, resulting in 751 cases of food poisoning (Török et al., 1997).

These incidents underscore the challenges of identifying and attributing bioweapon use, as well as the widespread public fear such attacks can provoke. Notably, they have primarily involved naturally occurring agents or those subjected to relatively minor modifications, such as engineering drug resistance. However, the future of biological and toxin weapons may deviate significantly from these historical patterns. Advances in biotechnology and artificial intelligence raise concerns that future threats could involve entirely novel agents designed for specific characteristics, such as enhanced transmissibility or pathogenicity, targeted effects on particular populations, or resistance to existing detection and countermeasure systems or existing immunity (Sandbrink, 2023; Drexel and Withers, 2024; Pannu et al., 2024). Such developments could render traditional preparedness strategies insufficient, highlighting the urgent need for proactive measures and adaptable biosecurity frameworks to address these emerging risks.

### Emerging challenges in the contemporary landscape

The modern era is witnessing a renewed awareness of the possibilities of biological warfare in light of rapid advancements in biotechnology, artificial intelligence, and shifting geopolitical dynamics (Juling, 2023; Brent et al., 2024; Berg and Kappler, 2024). These developments expand the potential scope and sophistication of potential threats, elevating biological weapons to a central concern in contemporary security discussions.

The rise of hybrid warfare—characterised by the integration of conventional military tactics with unconventional methods—further complicates the biological risk landscape. A biological weapons attack, for example, could be coordinated with cyberattacks targeting health infrastructure, undermining emergency response efforts, or paired with disinformation campaigns to sow public panic and distrust (Smith, 2019; Chatham House–International Affairs Think Tank, 2024). These strategies could amplify the impact of a biological assault, rendering traditional mitigation measures inadequate and necessitating more comprehensive, integrated security frameworks.

### Challenges in detecting and attributing malicious use

Detecting malicious intent in the development of biological weapons presents unique challenges, particularly when compared to other weapons of mass destruction. The raw materials and technologies required for bioweapons often overlap substantially with those used in legitimate fields, such as medical research, public health, and agriculture, complicating efforts to distinguish misuse from beneficial applications (Koblentz, 2009). AI is also likely to enable the design of novel sequences with pathogenic or toxic functions, challenging existing frameworks for detecting threats based on similarity to historical hazards (US National Science and Technology Council, 2024; U.S. HHS, 2023). Advances in laboratory automation and the self-replicating nature of biological agents further exacerbate these challenges, enabling rapid scale-up with minimal infrastructure. Additionally, the democratisation of synthetic biology tools, which are increasingly accessible to a global audience, reduces the technical and logistical barriers to bioweapon development (Lee et al., 2023). Together, these factors underscore the need for robust biosecurity frameworks to mitigate the dual-use risks of biotechnology (WHO, 2022a).

### The role of AI in shaping the biorisk landscape

AI has emerged as a potentially transformative factor reshaping the scope and sophistication of biological threats (Carter et al., 2023; Sandbrink, 2023; Drexel and Withers, 2024). In protein design and bioengineering, AI-driven tools could streamline the creation of bioweapons by enabling the design of proteins with tailored properties, such as enhanced heat stability, solubility, or binding specificity—traits that could increase their efficacy as weapons (Drexel and Withers, 2024; Watson et al., 2023; Sumida et al., 2024). For instance, AI can be used to develop novel proteins capable of binding to specific targets (Zambaldi et al., 2024), a capability with potential applications for toxins and biologics in military contexts. Beyond biological design, AI-powered systems like chatbots can enhance logistical aspects of bioweapons development, including planning, acquisition of materials, operational coordination, and delivery strategies (Soice et al., 2023; OpenAI, 2024).

AI systems can automate complex scientific tasks, reducing the need for advanced training or deep domain knowledge. For instance, AI can design experiments, optimise chemical or biological designs and synthesis pathways, and predict the efficacy of biological agents with reduced human oversight. This de-skilling effect democratises access to capabilities that were once restricted to well-funded state actors or specialised institutions, which may empower non-state actors or small groups to credibly develop sophisticated bioweapons (CLTR, 2024). For highly resourced actors, the possibilities are even more alarming, with AI opening avenues for designing novel toxins with more targeted and tunable effects, or the development of pathogens with impacts far exceeding those seen in nature. However, toxins without an effective self-replicating delivery tool function more similarly to chemical weapons, limiting their potential for mass harm, and experts remain divided on the feasibility of effective pandemic pathogen design (Carter et al., 2023; Pannu et al., 2024). As AI continues to advance, its dual-use potential underscores the urgent need for robust safeguards to prevent misuse while enabling beneficial scientific progress.

The credibility of the threat posed by AI-enabled bioweapons remains a contentious issue, with expert opinions varying widely across disciplines, including historical biological weapons (BW) programs, biotechnology, security, and artificial intelligence (Carter et al., 2023). Substantial testing has been conducted on large language models (LLMs) (OpenAI, 2024; Mouton et al., 2024; Phuong et al., 2024), and has yielded mixed findings. Early evaluations suggested minimal uplift compared to information readily available on the internet (Mouton et al., 2024). However, OpenAI’s o1 model demonstrated measurable benefits for experts, particularly in synthesising existing threat information and enhancing access to previously obscure knowledge (OpenAI, 2024).

No direct testing of the misuse potential of AI tools used in biological design (biological design tools, or BDTs) has been publicly reported. The most advanced of these capabilities is protein design tools, but this class of tools also includes those for designing DNA, biological circuits and cells (Carter et al., 2023). Key risks identified for protein tools include the design of novel toxins and effectors, the design of novel viral pathogens and vectors, and the alteration of existing pathogen proteins to change their properties in ways that increase their utility as weapons, such as host range, binding to target cells, and evasion of natural immunity. Indicators of emerging risks include advancements in targeted therapeutics, which reduce the number of iterations required to achieve successful designs, improvements in automation processes, and the increasing efficiency of mass production systems. These developments collectively suggest that BDTs could enable more rapid and scalable approaches to bioweapons design in the future. Addressing these gaps requires a focused effort to evaluate BDT risks rigorously, using realistic yet ethically sound testing methods. This includes incorporating benign use cases that reflect the operational challenges of weaponisation, as well as investing in the development of frameworks to detect and mitigate emerging threats.

### Implications for biosecurity

The changing biorisk landscape underscores the urgency of addressing the dual-use potential of AI in biotechnology. Policymakers, scientists, and industry leaders must recognise that traditional biosecurity frameworks are insufficient to counter emerging threats. Without proactive measures, the risk of AI exacerbating bioweapons development will continue to grow, posing serious threats to global security and public health. This evolving context necessitates a reimagining of biosecurity approaches, with discussions focusing not only on restricting access but also on promoting transparency, accountability, and innovation for defensive and beneficial purposes.

## Collaborative responses to AI risks in biotechnology

As the potential for misuse of AI in biotechnology becomes an increasing concern, policymakers, private sector stakeholders, and the scientific community have begun taking steps to address the associated risks. These efforts focus on fostering collaboration, developing safeguards, and implementing frameworks that balance innovation with biosecurity. This section examines the initiatives led by these groups and highlights the challenges and opportunities they present.

### Policy efforts

Policymakers across the globe are increasingly prioritising the risks posed by AI in biotechnology, particularly the potential for these technologies to lower barriers to developing bioweapons (AI Safety Summit, 2023; U.S. Department of Homeland Security, 2024). Recent high-profile events, such as the AI Safety Summit 2023, have brought biosecurity concerns to the forefront, highlighting the need for international collaboration and education (GOV.UK, 2023; GOV.UK, 2024a). These initiatives aim to inform policymakers about the technical complexities of AI-driven advancements in biotechnology and their dual-use implications. AI Safety Institutes are also forming in countries around the world to evaluate risks and inform policymaking (AISI, 2024; NIST, 2023; AISI Japan, 2024; Shaping Europe’s digital future, 2024).

Governments and international organisations are advancing frameworks to assess and mitigate the misuse potential of biotechnology tools. Efforts to improve DNA synthesis screening are a vital component of these efforts (Baker and Church, 2024). In the United States, a new policy mandates that nucleic acids purchased with federal research funding must come from providers that screen orders for potential misuse (US National Science and Technology Council, 2024; The White House, 2023). Similarly, the United Kingdom has introduced its first-ever guidance for domestic nucleic acid providers, outlining best practices for screening synthetic nucleic acid orders (GOV.UK, 2024b). The recent U.S. Executive Order on AI (The White House, 2023) established additional specific measures to evaluate and mitigate risks from AI-driven biotechnologies. Among these measures is a requirement for reporting on biological foundation models with a lower floating-point operations per second (FLOP) threshold compared to other AI applications, reflecting their heightened dual-use concerns. These initiatives mark significant progress in balancing the opportunities of AI-enabled biotechnology with the critical need for global biosecurity.

### Industry-led initiatives

Major AI companies are taking proactive steps to assess and mitigate the risks associated with their technologies. Participation in collaborative platforms like the Frontier Model Forum (Frontier Model Forum, 2024c) and AIxBio Global Forum (NTIbio, 2024) which bring together key stakeholders to share best practices, develop guidelines, and promote the safe deployment of tools has become a critical process for AI companies to share learnings and best practices and inform their internal policies (Frontier Model Forum, 2024a; Frontier Model Forum, 2024b). Companies like OpenAI (OpenAI, 2024), Meta (Dubey et al., 2024; Anthropic, 2024) and DeepMind (Grin et al., 2024) have also commissioned comprehensive safety evaluations for their models, often including red-teaming exercises designed to uncover vulnerabilities and identify potential misuse. However, these efforts demand careful oversight to prevent accidental disclosure of sensitive findings or unintended misuse. Some companies have placed their models behind an interface that allows them to control and monitor access, but others have released their models fully in the public domain, precluding the implementation of robust governance mechanisms. A new industry is forming around providing risk assessments and safety evaluations for AI and biotechnology applications. While best practices for this sector are still being developed, there is growing demand for experts in biotechnology to design robust safety protocols and capability benchmarks.

### Academic and research community efforts

The academic community has increasingly recognised its responsibility to mitigate biosecurity risks while continuing to advance scientific discovery. One significant step has been the protein design community’s issuance of a statement on the responsible use of AI in biodesign, which underscores the importance of ethical considerations in deploying these powerful tools (Responsible AI x Biodesign, 2024). In parallel, some research funders now require applicants to submit statements detailing how potential dual-use applications of their work, including bioweapons risks, will be mitigated (Wellcome, 2024). The Biofunders Compact has further encouraged bioscience and biotechnology funders to make public commitments to integrating biosecurity and biosafety into their funding decisions, promoting accountability and transparency (The Nuclear Threat Initiative. NTI, 2024b). Academic contributions to defensive measures have also been notable. Research into methods for detecting genetic engineering (Wang et al., 2019; Alley et al., 2020) and attributing the origins of engineered organisms (Wang et al., 2021), has advanced significantly. Progress in DNA synthesis screening technologies (Godbold et al., 2021; Wheeler et al., 2024), and microbial forensics (Inglis, 2024; Tripathi et al., 2024) further illustrates the research community’s proactive role in addressing dual-use risks. The academic community faces particular challenges in securely sharing sensitive results on the safety and security of AI capabilities without the inappropriate proliferation of dual-use information. Collectively, these efforts exemplify how the academic community is balancing the imperative of innovation with the need to ensure global biosecurity.

### Challenges and opportunities

While significant progress has been made in addressing the risks associated with AI in biotechnology, notable challenges remain. The demand for scientific expertise to support policymakers and international bodies, such as the Biological Weapons Convention (BWC) (The InterAcademy Partnership, 2024), continues to grow, placing pressure on the availability of knowledgeable advisors. Policymakers and the scientific community must also navigate the delicate balance between fostering innovation and implementing necessary regulations. Despite these challenges, the current landscape offers significant opportunities. Strengthening the biosecurity framework can foster interdisciplinary collaboration between AI developers, biologists, and policymakers, creating a more unified approach to managing dual-use risks.

## Safeguards for mitigating risks: current state, opportunities and challenges

Safeguards to mitigate risks in AI and biotechnology are evolving, yet significant gaps persist. While a range of potential safeguards have been proposed, such as refusal mechanisms, tiered access controls, and enhanced monitoring systems (The Nuclear Threat Initiative. NTI, 2024a), their application in AI tools specific to biotechnology remains largely uncharted. Many of these tools operate in a dual-use space, where their capabilities can advance both beneficial applications, like therapeutic development, and potential misuse, such as bioweapon design, creating substantial challenges in mitigating risks of misuse while harnessing their benefits.

### Data controls

Some model developers have taken proactive steps to withhold data they deem risky, such as certain pathogen genomes, from AI models. Examples include the exclusion of sensitive datasets in tools like ESM3 (Hayes et al., 2024) and Evo (Nguyen et al., 2024). However, the open nature of many AI models has allowed fine-tuning with restricted data, potentially undermining these precautions (PathoLM, 2024; Workman and LatchBio, 2024). Policy proposals to limit access to future pathogen genome data have also emerged (Carter et al., 2023; Maxmen, 2021), aiming to preempt misuse. These proposals are not entirely new (Committee on Genomics Databases for Bioterrorism Threat Agents and Board on Life Sciences, 2004), but have gained renewed urgency in the context of AI’s rapid development and the increasing democratisation of biotechnology. While these proposals have garnered support in some policy circles, they have been met with criticism from experts who warn that restrictions could impede scientific progress and global collaboration (Committee on Genomics Databases for Bioterrorism Threat Agents and Board on Life Sciences, 2004). The World Health Organization (WHO) emphasises that sharing pathogen genome data is crucial for preventing, detecting, and responding to epidemics and pandemics, as well as for monitoring endemic diseases and tracking antimicrobial resistance (WHO, 2022b). Moreover, some experts question whether excluding pathogen data from AI training would significantly limit the development of concerning capabilities, noting that design methodologies often rely on unrelated or widely accessible data (The Nuclear Threat Initiative. NTI, 2024a). This ongoing debate reflects the complex balance between biosecurity and the need for scientific openness.

### Built-in safeguards in AI tools

Built-in safeguards are a critical component of risk mitigation strategies for general-purpose AI tools, designed to prevent misuse and ensure responsible deployment. These safeguards include mechanisms such as refusal systems that block harmful or unethical requests and hard-coded constraints to limit specific high-risk functionalities. However, the implementation and effectiveness of these measures vary significantly across AI domains. For large language models (LLMs), extensive safeguards have been adopted. Systems such as ChatGPT (OpenAI, 2024) and Gemini (Google Cloud, 2024) feature refusal mechanisms that prevent responses to potentially harmful queries, including instructions for creating weapons or performing unsafe chemical reactions. Yet even these safeguards can be stripped out if sufficiently open access is provided to the models, such as through the open release of model weights.

In contrast, the adoption of built-in safeguards for biotechnology-focused AI tools remains underdeveloped, despite their dual-use potential (The Nuclear Threat Initiative. NTI, 2024a). For example, AlphaFold3 were early adopters of experimental refusal mechanisms to block misuse, but these efforts were preliminary and highlighted the challenges of balancing functionality with security (Grin et al., 2024). The appropriateness of implementing refusal mechanisms in biological design tools remains a complex and underexplored issue. Unlike in large language models, where refusals can effectively block queries with clear malicious intent, biological design often resides in a dual-use space. Many therapeutic and diagnostic efforts inherently involve predictions and designs that overlap with weaponisation potential. For instance, the development of treatments for infectious diseases may require modelling pathogenic structures or engineering highly specific proteins, which could also be repurposed for harmful applications (Thadani et al., 2023). This overlap makes it challenging to delineate legitimate use cases from misuse solely through automated refusal systems (The Nuclear Threat Initiative. NTI, 2024a). Therefore, refusal mechanisms, if overly restrictive, could impede critical research, such as designing countermeasures for bioterrorism or creating synthetic vaccines. Balancing the need for accuracy and functionality in therapeutic and diagnostic efforts with biosecurity safeguards will require a nuanced approach, combining automated mechanisms with human oversight and contextual analysis, which will inevitably raise difficult trade-offs in promoting beneficial science, preventing harm, and financing the resources required for safety measures. This highlights the importance of further investigation into refusal mechanisms tailored to the unique challenges of biological design tools.

### Managed access frameworks

Open-weight models are highly valued by academic and research communities for their ability to foster transparency, reproducibility, and innovation. By allowing researchers to replicate studies, extend existing work, and democratise advanced technologies, these models have become indispensable tools in scientific progress. However, their open-access nature introduces significant risks of misuse, particularly in fields like biotechnology where dual-use applications can enable harmful purposes (The Nuclear Threat Initiative. NTI, 2024a).

Efforts to restrict access to open-weight models frequently encounter resistance from the academic and open-source communities. Advocates of open access argue that transparency is essential for scientific integrity, promoting collaboration, and accelerating innovation (Anonymous, 2024). This tension is further compounded by the rapid emergence of open-source versions of closed models, driven by demand for their functionality (Callaway, 2024). As a result, restricting access often proves challenging, as it may lead to the proliferation of unofficial and less-regulated alternatives.

Managed access frameworks offer a potential solution to these challenges by enabling the controlled distribution of AI tools while maintaining some degree of accessibility. Platforms like Together AI (2024) and Huggingface (2024) provide repositories and APIs that balance openness with accountability by requiring users to comply with ethical guidelines or community standards. Kaggle (2024), a popular platform for data science competitions, exemplifies another managed-access approach by providing datasets, models and computational resources in a controlled environment. These frameworks promote responsible use while still democratising AI tools. However, managed-access frameworks come with caveats. They would demand substantial resources for implementation and oversight, including providing cloud computing resources, monitoring usage, vetting users, and enforcing compliance. A key component of managed-access frameworks for biosecurity may involve Know Your Customer (KYC) processes, which are widely used in industries like finance to verify user identities. Applying KYC principles to AI access might include identity verification, institutional affiliation checks, and risk assessments of intended use. Such measures could leverage existing frameworks and technologies to enhance security while preserving accessibility. Using ORCIDs (Open Researcher and Contributor IDs) (ORCID, 2024) as part of a managed-access framework offers an efficient and scalable way to implement KYC principles in the life sciences. ORCIDs provide a persistent, unique identifier for researchers, allowing platforms to verify users' identities and affiliations while minimising administrative burdens. By integrating ORCIDs into access protocols, AI developers and data providers could ensure that only authenticated researchers with credible affiliations gain access to sensitive tools or datasets. This approach leverages an existing, widely adopted system, reducing barriers to implementation while enhancing accountability and traceability in the use of dual-use technologies.

Without adequate resources, these managed access systems risk being bypassed or failing to address security concerns comprehensively, and even with sufficient resources, they cannot eliminate the possibility of an “inside threat” who passes the various vetting requirements but still has nefarious intent.

### Evaluations and red teaming

Effective evaluations and red-teaming efforts are vital for advancing AI applications in biotechnology responsibly, yet the current landscape is hindered by inconsistent benchmarks, making it challenging to measure progress or compare capabilities across tools. Treaty commitments (UNODA, 2024) and ethical considerations often restrict direct assessments of harmful applications, leaving a critical gap in standardised testing protocols and evidence-based evaluations. Benign proxy tasks can shine light on capabilities of concern and the effectiveness of safeguards while avoiding the creation of harmful products (RAND, 2024). However, there is still ongoing debate about which proxy tasks are most effective for assessing risks related to biological weapons.

DiEuliis et al. (2024) highlight the need for robust and multifaceted approaches to evaluating biosecurity risks. Experts in biotechnology are needed to engage in red-teaming exercises, where participants simulate misuse scenarios to uncover vulnerabilities and inform mitigation strategies. They are also needed to feed in to ongoing, dynamic assessments of factors like technological readiness, accessibility, and the expertise required for potential exploitation of a tool. A particularly pressing need exists for benchmarks to evaluate design capabilities. Reliable metrics for assessing the efficacy and safety of AI-driven design tools are scarce, leaving gaps in understanding how these systems might be leveraged for dual-use purposes and presenting opportunities for research and engagement from the biotechnology community.

### Capture of design metadata

Capturing and standardising activity from DNA and protein sequence design tools offers a significant opportunity to enhance both the traceability and accountability of biological work. By cataloging the design process, including the steps taken and decisions made, researchers can create a transparent audit trail that not only strengthens biosecurity but also fosters trust and collaboration within the scientific community (The Nuclear Threat Initiative. NTI, 2024a). Such audit trails would be invaluable for DNA synthesis providers, enabling them to better assess the intent behind novel sequences submitted for synthesis. By understanding the design process, providers could more effectively evaluate potential risks and ensure compliance with biosecurity standards. Additionally, these records could serve as an important resource for publishing the methods used in scientific research, offering reproducibility and clarity in peer-reviewed studies. Moreover, sharing standardised data on sequence design could promote best practices in DNA and protein engineering, creating a foundation for collaborative innovation while mitigating risks of misuse. This approach aligns with efforts to balance the need for transparency and openness in research with the imperative of safeguarding against the dual-use potential of emerging biotechnologies.

### Current limitations and pathways for enhancement

The dual-use nature of AI in biotechnology raises urgent and complex questions about the effectiveness and consequences of proposed safeguards. For instance, could refusal mechanisms effectively block harmful applications without obstructing critical research? Might managed access models balance the need for security with the imperative of fostering open collaboration? Furthermore, what are the logistical and financial implications of implementing these safeguards at scale, especially for smaller institutions or researchers in resource-constrained settings?

Addressing these challenges will require a concerted effort involving rigorous testing of safeguard mechanisms, broad stakeholder engagement, and the creation of context-specific frameworks tailored to the unique risks and opportunities in biotechnology. Central to these efforts is the active participation of the biotechnology research community. Researchers must help to ground risk assessments in demonstrated and realistic future capabilities, ensuring that mitigation strategies are both effective at reducing risks and compatible with enabling beneficial research. By striking this balance, the community can help ensure that AI in biotechnology advances responsibly, maximising its potential for global good while minimising the risk of misuse.

## Discussion

The dual-use nature of AI in biotechnology underscores the delicate balance between fostering innovation and implementing safeguards. Over-regulation risks stifling progress, particularly in areas like therapeutic discovery and synthetic biology, where access to advanced tools can drive breakthroughs. On the other hand, insufficient safeguards leave the door open to potential misuse, from bioweapon development to accidental creation of harmful agents. Striking this balance requires policies that are flexible enough to adapt to evolving technologies while robust enough to address emerging threats. A proactive, interdisciplinary approach is essential to address the challenges posed by AI in biotechnology. Engagement between AI researchers, bioengineers, and security experts can foster a deeper understanding of dual-use risks and enable the development of practical safeguards.

A proactive approach, modelled on the success of the Asilomar Conference on recombinant DNA (Grace, 2015), can set the stage for responsible innovation in AI-powered biotechnology. Involving diverse stakeholders early in the conversation ensures that safety and ethical concerns are addressed without stifling progress. Unlike early genetic engineering efforts, current AI applications must prioritise addressing dual-use risks, including potential misuse for bioweapons or harmful applications. Effectively communicating the risks and benefits of AI in biotechnology is critical to building public trust. Simplifying complex issues without oversimplifying their implications can bridge the gap between experts and non-experts. By learning from the successes and challenges of regulating genetic engineering, stakeholders in AI-powered biotechnology can develop more effective and balanced governance frameworks, fostering innovation while minimising risks.

The biotechnology community must recognise its responsibility in this shared effort. Developing skills in public engagement, policy advocacy, and risk assessment is more critical than ever. If biotechnology is regulated entirely from the outside—without input from those who understand its complexities—it risks being shaped by poorly informed policies that hinder progress. At the same time, the community must take its responsibility seriously, prioritising safety and ethical considerations in every stage of research and development. The consequences of failing to act responsibly could jeopardise both public trust and the future of innovation in this transformative field.
